# Increased reports of measles in the Metropolitan City of Milan, northern Italy, September 2023 to March 2024

**DOI:** 10.2807/1560-7917.ES.2024.29.16.2400201

**Published:** 2024-04-18

**Authors:** Maria Gori, Clara Fappani, Silvia Bianchi, Sabrina Senatore, Daniela Colzani, Priscilla Pasutto, Melissa Baggieri, Silvia Gioacchini, Antonella Marchi, Paola Bucci, Emilio D’Ugo, Marino Faccini, Danilo Cereda, Luigi Vezzosi, Elisabetta Tanzi, Fabio Magurano, Antonella Amendola

**Affiliations:** 1 Department of Health Sciences, Università degli Studi di Milano, Milan, Italy; 2 Coordinated Research Centre “EpiSoMI”, Università Degli Studi di Milano, Milan, Italy; 3 Department of Clinical Sciences and Community Health, Università degli Studi di Milano, Milan, Italy; 4 Health Protection Agency of the Metropolitan Area of Milan, Milan, Italy; 5 Department of Infectious Diseases, Istituto Superiore di Sanità, Rome, Italy; 6 General Directorate of Welfare, Regione Lombardia, Milan, Italy; * These authors contributed equally to the work and share first authorship.

**Keywords:** measles surveillance, crossing-border, measles resurgence

## Abstract

Since late 2023, the Metropolitan City of Milan and surrounding areas (northern Italy) have been experiencing a resurgence of measles, with most cases detected starting from January 2024. During this brief period, we observed measles in travellers from endemic areas, participants in international events, vaccinees and healthcare workers. Indigenous cases have also been identified. Even though we have not yet identified large and disruptive outbreaks, strengthening surveillance and vaccination activities is pivotal to help limit the impact of measles spread.

Since 1 January 2023, the World Health Organization (WHO) European Region countries have documented an alarming resurgence of measles, showing an increase of over 30 times compared with the previous year [1], with large or disruptive outbreaks in Romania, Austria and France [2]. Italy also reported a significant measles resurgence, with 31 confirmed cases between September and December 2023 and 181 between 1 January and 31 March 2024 [3].

As Subnational Reference Laboratory of the Italian measles and rubella surveillance network MoRoNet [4] in the Metropolitan City of Milan and surrounding areas (nearly 4 million inhabitants, Lombardy region, northern Italy), we observed that measles activity began to increase in September 2023 after a period of low activity (August 2019-August 2023). Here we report the latest updates on measles resurgence in the area under surveillance.

## Latest updates on measles cases

**Figure 1:**
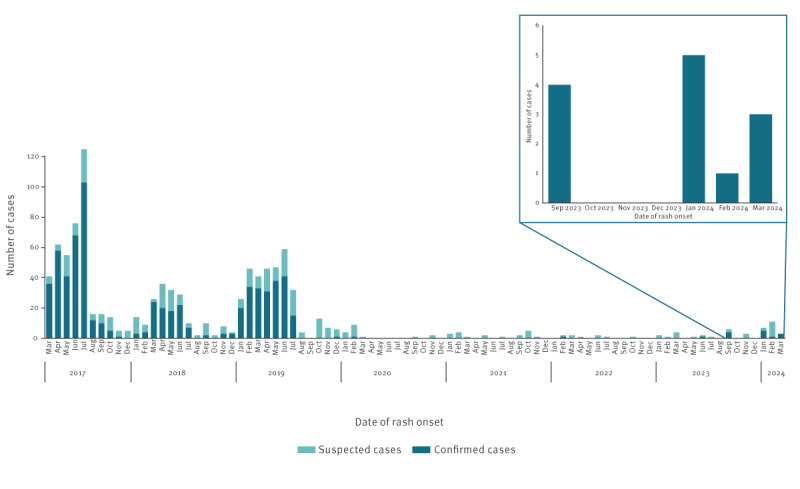
Suspected (n = 1,010) and confirmed (n = 669) measles cases in the Metropolitan City of Milan and surrounding areas, Italy, March 2017−March 2024

We investigated 30 suspected measles cases between September 2023 and March 2024, 13 of which were confirmed through the detection of virus-specific IgM antibodies in serum by enzyme-linked immunosorbent assay, and measles virus (MeV) RNA in paired urine and oropharyngeal swabs samples by real-time retrotranscription (RT) PCR [5]. Notably, nine of the 13 confirmed cases were detected between January and March 2024 (**Figure 1**).

The median age of the 13 confirmed cases was 33 years (range: 1–49 years), and eight cases were hospitalised. Two cases were fully vaccinated, and one case received one dose of measles-containing vaccine (**Table**). Eleven of the 13 confirmed cases had a history of travel (to Indonesia, Malaysia, India, Uzbekistan, Thailand and two to other regions in Italy) or were linked to travel-related cases. In detail, Case 2 returned from Malaysia and attended the same business meeting as Case 3, Case 10 was a fully vaccinated healthcare worker, likely infected in the emergency department where Case 9, a returning traveller from Thailand, was admitted.

**Table:** Confirmed measles cases in the Metropolitan City of Milan and surrounding areas, Italy, September 2023−March 2024 (n = 13)

Case ID	Date of rash onset	Vaccination status	Hospitalisation	Genotype	MeaNS2 DSId	Source	Sequence name
1	7 Sept 2023	Yes, 1 dose	Yes	D8	8383	Travel (Indonesia or Singapore)	MVs/Milano.ITA/36.23
2	8 Sep 2023	No	Yes	D8	8477	Travel (Malaysia)	MVs/Milano.ITA/36.23/2
3	20 Sep 2023	No	Yes	D8	8477	Travel-spread (linked to Case 2)	MVs/Milano.ITA/38.23/2
4	21 Sep 2023	Yes, 2 doses	No	D8	2279	Travel (India)	MVs/Milano.ITA/38.23
5	6 Jan 2024	No	No	D8	8581	Travel (Uzbekistan)	MVs/Milano.ITA/1.24
6	15 Jan 2024	No	Yes	D8	8491	Travel (Italy)	MVs/Milano.ITA/3.24
7	19 Jan 2024	No	Yes	D8	2279	Indigenous	MVs/Milano.ITA/3.24/2
8	19 Jan 2024	No	Yes	D8	2279	Indigenous	MVs/Milano.ITA/3.24/3
9	23 Jan 2024	No	Yes	D8	8491	Travel (Thailand)	MVs/Milano.ITA/4.24
10	6 Feb 2024	Yes, 2 doses	No	D8	8491	Travel-spread (linked to Case 9)	MVs/Milano.ITA/6.24
11	12 Mar 2024	No	Yes	D8	8491	Travel (Italy)	MVs/Milano.ITA/11.24
12	12 Mar 2024	No	No	D8	8491	Travel (Italy)	MVs/Brescia.ITA/11.24
13	12 Mar 2024	No	No	D8	8491	Travel (Italy)	MVs/Milano.ITA/11.24/2

MeaNS2: World Health Organization Measles Virus Nucleotide Surveillance; DSId: distinct sequence identifier.

At the time of writing, we have confirmed three cases (Cases 11, 12 and 13) returning from the same international exhibition held in northern Italy in week 9 2024.

## Genotyping and phylogenetic analysis

**Figure 2:**
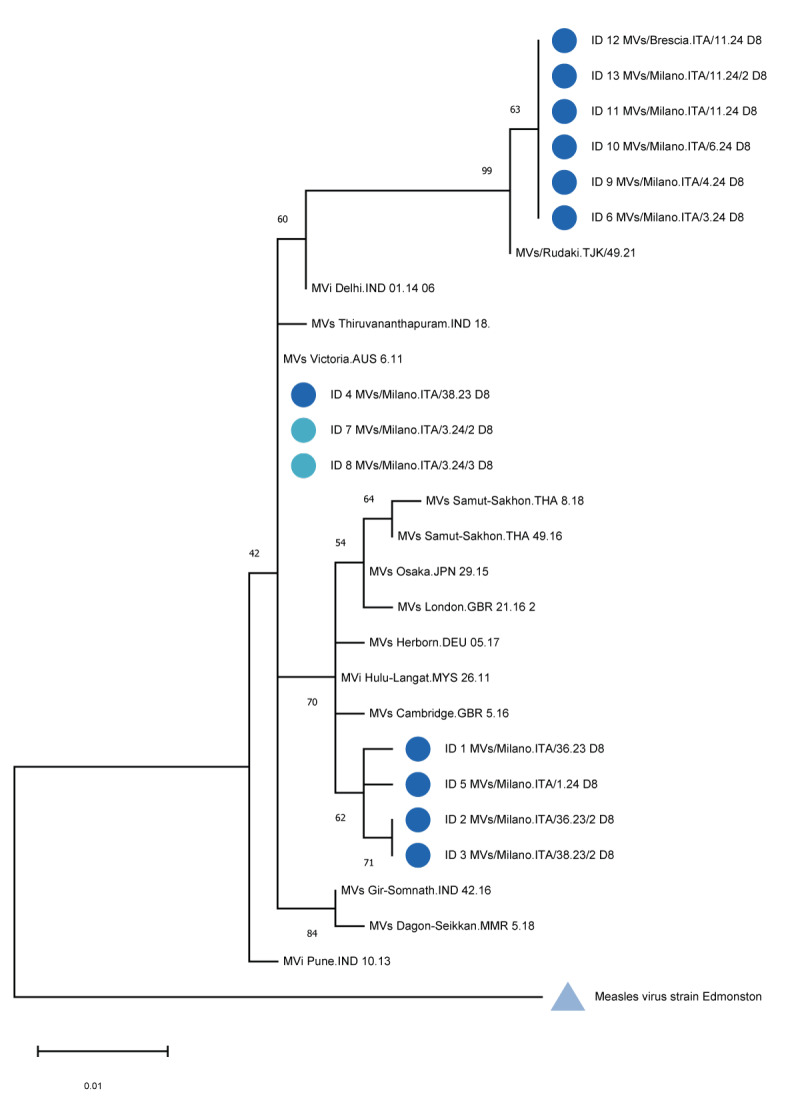
Phylogenetic analysis of measles isolates identified in the Metropolitan city of Milan and surrounding areas, Italy, September 2023−March 2024 (n = 13)

Isolates from the confirmed cases were successfully genotyped by amplifying and sequencing the highly variable 450 nucleotide (nt) region in the C-terminal of the nucleoprotein (N-450) according to the WHO manual [5]. All sequences were classified as genotype D8 (**Figure 2**).

The tree was constructed using genotype A strain MVs/Vaccine/Edmonston as outgroup (grey triangle).

Evolutionary analyses were conducted in MEGA11 [13]. The evolutionary history was inferred by using the Maximum Likelihood method based on the Kimura 2-parameter model [14]. Branch support (1,000 bootstrap iterations [15]) is provided next to nodes. The tree with the highest log likelihood (-918.56) is shown. The percentage of trees in which the associated taxa clustered together is shown next to the branches. Initial tree(s) for the heuristic search were obtained automatically by applying Neighbour-Join and BioNJ algorithms to a matrix of pairwise distances estimated using the Maximum Composite Likelihood (MCL) approach, and then selecting the topology with superior log likelihood value. The phylogenetic tree includes sequences from travel-related cases and linked to travel-related cases (blue circles) and indigenous cases (light blue circles).

All isolates were submitted to the GenBank sequence database (https://www.ncbi.nlm.nih.gov/genbank/) [6] (accession numbers: PP334141–PP334142 and PP556752–PP556762) and deposited in the WHO Measles Virus Nt Surveillance database (MeaNS2, https://who-gmrln.org/means2) [7]. Five different Distinct Sequence Identifiers (DSId) were assigned (8383, 8477, 2279, 8581, 8491). Notably, six sequences were identical and belonged to a previously described variant, namely DSId 8491 [8,9].

## Discussion

Prior to the COVID-19 pandemic period, we observed three epidemic peaks in the Metropolitan City of Milan and surrounding areas: March–June 2017, April–June 2018 and January–July 2019 [10]. Since August 2019, we noticed a significant drop in measles cases with only four confirmed cases of 86 suspected as of August 2023, none of which was autochthonous.

Those affected between September 2023 and March 2024 were mostly adults and young adults; two cases were fully vaccinated and one case received one dose of measles-containing vaccine.

To note, we noticed an increase in the number of measles cases after the holiday seasons, in September 2023 and January 2024. However, since January 2024, we have continued to observe measles cases. Almost all confirmed cases had a history of travel or were linked to travel-related cases. We have also observed two non-travel-related cases (Cases 7 and 8, referred to as indigenous), which had not been detected since 2019. Although these two cases were affected by the same DSId 2279 and lived north-west of Milan, no epidemiological correlation was found, suggesting the presence of underreported cases in mid-January 2024. In the aftermath, a provision of the Italian regional health authorities raised the attention on measles surveillance, investigating all cases with fever and rash (Regional regulation n. G1.2024.0004194, 6 February 2024).

Overall, we observed five different genotype D8 DSIds from Asia and from two other Italian regions, suggesting multiple introductions of MeV; however, in contrast to outbreaks that occurred between 2017 and 2019 [10,11], no onward transmission was observed so far. This could be due to the major effort of the competent authorities to improve vaccination coverage and recommend vaccination in fragile populations, travellers and healthcare workers (Regional regulation n. G1.2024.0004194, 6 February 2024). Indeed, the Lombardy Region currently has achieved 96% vaccination coverage of the first dose of measles-containing-vaccine and 93% for the second dose (unpublished data).

Particular attention should be paid to DSId 8491 that was the most frequent variant, being detected in six cases. The first case affected with this variant was identified in Italy in mid-January [9] and was also responsible for an outbreak in the Federation of Bosnia and Herzegovina between December 2023 and February 2024 [12]. At the time of writing (March 2024) we have observed three cases (Cases 11, 12 and 13) who had attended the same international exhibition, which may represent a potential superspreading event. The Italian national health authorities were promptly alerted. These cases were affected by the DSId 8491 variant. Importantly, it has been demonstrated that this D8 variant can be detected with reduced sensitivity by some currently used diagnostic tests [8].

The probability of exposure to MeV is expected to rise in the coming weeks due to the typical seasonal pattern of the virus [2].

Of note, the area under surveillance represents the most important Italian connection to central and northern Europe, with tourist, migratory and business mobility of people, which increases the risk of importation as well as exportation of measles between countries.

## Conclusion

Measles is on the rise in the Metropolitan City of Milan and surrounding areas, mainly among adults and also involving vaccinated individuals and healthcare workers. Even though we have not yet identified large and disruptive outbreaks, strengthening fever and rash surveillance and catch-up vaccination activities is pivotal to help limit the impact of cross-border transmission following travels to endemic countries, and facilitate control of new clusters.
